# Notes on Preparations for the Sick

**Published:** 1898-09

**Authors:** 


### Notes on Preparations for the Sick.

**Antikamnia and Salol Tablets.**—JOHN MORGAN RICHARDS, London.—This combination hails from the United States. The coal-tar product called Antikamnia is now well known both in this country and in America from the oft reiterated advertisement that it "does not depress the heart." We were for a long time of the opinion that its negative quality was its only virtue, but an increased experience has shewn that it possesses positive anodyne and hypnotic powers, which, taken in conjunction with salol, may well justify its claim to be antirheumatic, antipyretic, and analgesic.

**Palatinoids: Thyroid Gland; Red Bone Medulla; Creasote; Amyl Nitris; Lapactic. Bipalatinoids: Creasote with Compound Hypophosphites.**—OPPENHEIMER, SON & CO., LTD., London.—The palatinoid is a very useful form of administration of **Thyroid Gland** and **Red Bone Marrow**, which have established themselves as necessary elements in the treatment of a variety of conditions. The red bone marrow has proved itself to be of use in the graver forms of anæmia, more particularly when the arsenical treatment cannot be continued. The purified beech-wood **Creasote** which is used in these palatinoids has no corrosive action on the gastric or buccal mucous membrane. The drug can be given in a tasteless and odourless form, each palatinoid having from half a minim to three minims. A dose of 15 minims daily is easily reached and continued indefinitely without nausea. The **Nitrite of Amyl** palatinoids are made in the strength of one, two, or three minims. The envelope appears to retain this volatile drug remarkably well, and the contents when allowed to escape by cutting open the capsule have their usual potent characteristics. The **Lapactic** palatinoid is the combination of aloin (gr. $\frac{1}{4}$), strychnine (gr. $\frac{1}{60}$), ext. belladonn. (gr. $\frac{1}{8}$), and ipecac. (gr. $\frac{1}{16}$) which the late Sir Andrew Clark so much commended. It should at least be as useful as pill or tabloid.

The bipalatinoid combination of creasote or guaiacol with hypophosphites is very useful in the treatment of phthisis. It may be said that the dose is small (♏ss. to j.), and hence it is not possible to give full doses of ten to twenty minims of guaiacol in this way; but as an occasional variation from the ordinary routine we have found these bipalatinoids to be highly acceptable to the patient, and there are times when full doses of the creasote drug are not desirable.

---

**Glandulen.**—THOMAS CHRISTY & Co., London.—Dr. Hofmann, of Meerane in Saxony, manufactures this product from the bronchial glands of freshly killed sheep. Each tablet is equivalent to 3½ grains of the fresh gland-substance. A dose of one to five tablets three times daily has entirely realised the results anticipated. Several medical men have expressed approval of this treatment, but we think it will require a large amount of evidence to establish the statement that the drug is "an organic preparation with a specific action in tuberculosis of the lungs."

---

**Ideal Milk.**—ANGLO-SWISS CONDENSED MILK CO. LIMITED, London.—This excellent preparation is well suited for use in certain ailments of the alimentary canal. It is digested easily, has a pleasant taste, and is not overloaded with sugar as some preparations of this kind are. We can recommend it.

---

**Panopepton; Pepsencia; Peptogenic Milk Powder.**—FAIRCHILD BROS. & FOSTER, New York.—These Fairchild preparations are the result of persistent efforts to produce the most useful forms of digestive ferments suitable for the predigestion of food for the sick and for the infant.

The **Panopepton** is the entire edible substance of lean beef and wheat flour, cooked, digested, sterilised, and concentrated *in vacuo*. It gives no precipitate with heat or nitric acid, and it is a clear fluid with no sediment of starch or fibre: both the albuminous and the amylaceous components have therefore been completely digested, and analysis shews that peptones and saccharine products are present, as the fluid gives the characteristic reaction for peptone with Fehling's fluid and with picric acid, and it reduces the Fehling's fluid on boiling. The specific gravity of the fluid is approximately 1060. There is nothing unpleasant in the taste of this peptone solution: its flavour somewhat resembles that of roast beef juice and crust of bread. A wineglassful of panopepton, with a biscuit or cracker, is suggested to the brain-worker when too tired for the digestion of ordinary foods. For many cases of insomnia it has proved itself to be a useful resource. For invalids when travelling it is a convenient food always ready. In many other conditions it may be relied upon to replace milk, as it affords all the principles necessary for the complete nutrition of the body. Messrs. Fairchild Bros. & Foster state that "for many years the peptonisation of a combined beef and wheat food has been the subject of constant experiment and study;" it must be a source of gratification to them to know that their efforts seem now to be successful, and are likely to be increasingly appreciated by both physician and patient.

The **Pepsencia** is a solution of the essential organic ingredients of the gastric juice, extracted directly from the peptic glands of the stomach of the calf. It is suggested that its principal value is for three distinct purposes: as a remedy for indigestion in adults and infants, as a means of administering drugs which disturb the digestive functions and impair the appetite, and as a practical rennet agent.

The **Peptogenic Milk Powder** yields a food for infants which is almost identical with human milk, and will afford a complete substitute during the entire nursing period. It has been found to be a most successful food for sick and feeble infants, as well as for adults whose digestion is enfeebled from any cause.

---

**Scotch Cream Whisky.**—R. H. THOMSON & Co., Leith.—This brand of Scotch whisky bears on its label the descriptive epithets "real and rare," "very old," and "guaranteed pure." Its flavour and the absence of after-effects seem to justify these appellations.

---

**Soloids: Eucaine and Cocaine; Boric Acid; Potassium Chromate.**—BURROUGHS, WELLCOME & Co., London.—Each of the first-named of these soloids contains ½ grain of eucaine hydrochloride and ½ grain of cocaine hydrochloride, and when dissolved in 22 minims of water makes a 5 per cent. solution which is a perfect local anæsthetic. Always studying the convenience of actual or possible clients, this firm now sends out soloids each of which contains 15 grains of boric acid. The advantages of these are obvious. The last-named of these preparations form part of a series of reagents required for water analysis, and are intended for observations outside the laboratory. The portability of reagents in this shape, and the ease with which definite quantities can be used as required, will recommend the soloids to those who practise field observations of this kind.

---

**Surgical Shirt; Surgical Gown.**—MARIA J. WEBB, Bristol.—These garments are intended to facilitate the application of massage, and also to take the place of the ordinary bed-gown in patients who have undergone operations about the thorax, and are especially designed for ladies who have had excision of the mammary gland. We greatly doubt if, for the latter purpose, they will meet with a favourable reception from surgeons or nurses. They are unnecessarily encumbered with buttons, and the absence of any gusset in the armpit would prevent their use in cases where a dressing was applied to the axilla.

